# Neuronal pentraxin receptor-1 is a new cerebrospinal fluid biomarker of Alzheimer’s disease progression

**DOI:** 10.12688/f1000research.15095.1

**Published:** 2018-07-05

**Authors:** Ilijana Begcevic, Magda Tsolaki, Davor Brinc, Marshall Brown, Eduardo Martinez-Morillo, Ioulietta Lazarou, Mahi Kozori, Fani Tagaraki, Stella Nenopoulou, Mara Gkioka, Eutichia Lazarou, Bryant Lim, Ihor Batruch, Eleftherios P. Diamandis

**Affiliations:** 1 Department of Laboratory Medicine and Pathobiology, University of Toronto, Toronto, Canada; 2 Department of Pathology & Laboratory Medicine, Mount Sinai Hospital, Toronto, Canada; 3 1st Department of Neurology, Medical School, Aristotle University of Thessaloniki, Thessaloniki, Greece; 4 Department of Clinical Biochemistry, University Health Network, Toronto, Canada; 5 Department of Biostatistics, Fred Hutchinson Cancer Research Center, Seattle, WA, USA; 6 Laboratory of Medicine, Department of Clinical Biochemistry, Hospital Universitario Central, Oviedo, Spain

**Keywords:** Alzheimer’s disease, biomarkers, cerebrospinal fluid, mass spectrometry, selected reaction monitoring, neuronal pentraxin receptor-1, Alzheimer’s disease progression, dementia

## Abstract

**Background: **Alzheimer’s disease (AD) is the most common type of dementia, with progressive onset of clinical symptoms. The main pathological hallmarks are brain deposits of extracellular amyloid beta plaques and intracellular neurofibrillary tangles (NFT). Cerebrospinal fluid reflects pathological changes in the brain; amyloid beta 1-42 is a marker of amyloid plaques, while total and phosphorylated tau are markers of NFT formation. Additional biomarkers associated with disease pathogenesis are needed, for better prognosis, more specific diagnosis, prediction of disease severity and progression and for improved patient classification in clinical trials. The aim of the present study was to evaluate brain-specific proteins as potential biomarkers of progression of AD.

**Methods: **Overall, 30 candidate proteins were quantified in cerebrospinal fluid (CSF) samples from patients with mild cognitive impairment (MCI) and mild, moderate and severe AD dementia (n=101) using mass spectrometry-based selected reaction monitoring assays. ELISA was used for neuronal pentraxin receptor-1 (NPTXR) confirmation.

**Results: **The best discrimination between MCI and more advanced AD stages (moderate and severe dementia) was observed for protein NPTXR (area under the curve, AUC=0.799). A statistically different abundance of this protein was observed between the two groups, with severe AD patients having progressively lower levels (p<0.05). ELISA confirmed lower levels in AD, in a separate cohort that included controls, MCI and AD patients.

**Conclusions: **We conclude that NPTXR protein in CSF is a novel potential biomarker of AD progression and could have important utility in assessing treatment success in clinical trials.

## Introduction

Alzheimer’s disease (AD) is a neurodegenerative disease characterized by progressive cognitive decline, behavioural problems and impairment of daily living activities. The main pathological hallmarks of AD are brain extracellular deposits known as amyloid β (Aβ) plaques, composed of aggregated Aβ fragments and intracellular neurofibrillary tangles (NFT), containing hyperphosphorylated protein tau (p-tau) fibrils ^1^. The diagnosis of AD is currently made based on core clinical criteria, including medical history, mental status testing, and neurological and physical assessment ^2^. With such criteria, only probable dementia due to AD can be diagnosed, while definitive diagnosis of AD can be made only post mortem, by neuropathological examination of different brain regions. More recently, a blood test for AD diagnosis has been suggested but it has not as yet been clinically validated ^3^. Three stages of AD have been recognized by the National Institute on Aging (NIA) and the Alzheimer’s Association newly revised diagnostic and research criteria: preclinical stage, mild cognitive impairment (MCI) due to AD, and dementia due to AD ^4^. Preclinical stage describes asymptomatic individuals with existing early brain pathology, while MCI due to AD includes patients with prodromal, mild symptoms as a result of disease pathology. Patients with dementia due to AD have impaired memory, thinking and behavioural functions, accompanied by severe pathological brain changes. Clinical symptoms typically appear gradually, indicating different levels of dementia severity: mild dementia (or early stage), moderate dementia (or middle stage) and severe dementia (or late stage) ^5^.

Cerebrospinal fluid (CSF) is a proximal fluid of the central nervous system, residing in direct contact with the brain parenchyma and thus can reflect physical and pathological changes in the brain ^6^. As such, CSF may be the most promising source of AD biomarkers; especially highly specific, brain-related protein biomarkers. The best evaluated AD biomarkers to date are CSF Aβ1-42, total tau (t-tau) and p-tau levels ^7^. These core AD biomarkers reflect main pathological hallmarks: Aβ1-42 peptide is a marker of Aβ plaque formation, while t-tau and p-tau are biomarkers of neuronal injury. Decreased CSF levels of Aβ1-42 and increased levels of t-tau and p-tau have been observed in AD patients, compared with healthy controls ^8^. Still, these extensively studied biomarkers are not widely used in the clinic, largely due to the lack of method standardization, and are mostly utilized in research settings, as also suggested by the new AD diagnostic guidelines ^2^. In addition, current CSF biomarkers have been tested in clinical trials of different AD therapeutic approaches. The results were contradictory, questioning their usefulness as indicators of efficacy of new therapies ^9^. It has also been previously reported that current AD biomarkers do not correlate well with cognitive decline in AD patients ^10, 11^.

There is a clinical need for novel biomarkers of AD progression. Such biomarkers could accurately and proactively identify evolving cases of AD and could be invaluable in clinical trials for patient enrichment and/or as surrogate endpoints. Moreover, such biomarkers could contribute to the better understanding of the underlying pathological mechanisms of AD.

Differential expression of proteins specific to a particular tissue can have strong disease specificity, pinpointing to pathology unique to that tissue. Some of these tissue-specific proteins have already shown promise as potential biomarkers, such as in male infertility (testis-specific protein TEX101) and in cerebral hemorrhagic stroke (brain-specific proteins NFM, α-Inx and β-Syn) ^12, 13^. In our recent study, we identified a set of brain-specific proteins that are consistently detected in the normal CSF proteome ^14^. The brain-specific proteins were retrieved from the Human Protein Atlas (HPA) tissue-specific database ^15^ and encompassed tissue-enriched (mRNA expression at least five times higher in the particular tissue (i.e. brain) relative to other tissues) and group-enriched proteins (mRNA expression at least five times higher in the group of 2–7 tissues (including brain), relative to all other tissues). These proteins were also secreted and/or were membrane-bound (as defined by HPA). We have further developed targeted mass spectrometry-based assays for quantification of 30 of these highly specific brain proteins in CSF ^16^. The main objective of the present study is to evaluate these 30 brain-related proteins for their ability to differentiate various stages of AD severity, i.e. MCI, mild, moderate and severe AD dementia, by utilizing state-of-the-art mass spectrometry-based selected reaction monitoring (SRM) assays. Considering that the apolipoprotein E (APOE) ε4 allele is the strongest genetic risk factor for developing AD, associated with disease pathology ^17^, we have further evaluated if the abundance of our candidates was related to the APOE phenotypes.

## Methods

### Multiplex selected reaction monitoring

A multiplexed, scheduled, SRM assay was developed for 30 brain-related proteins and is described in detail elsewhere ^16^. A protein previously found not to change in AD CSF (extracellular matrix protein 1, ECM1) was included as a negative control. Also included was a protein primarily related to demyelinating diseases (myelin basic protein, MBP). The SRM method for MBP has been described elsewhere ^13^. A peptide corresponding to apolipoprotein B (APOB) protein (a plasma protein) was also monitored, to check for blood contamination ^18^. For peptides containing methionine, both oxidized and non-oxidized forms of the peptide were monitored. Four peptides that represent different APOE phenotypes where additionally added to the assay, including an APOE peptide for total APOE, as a control. The APOE method was previously published ^18^.

### Mass spectrometry sample preparation

CSF samples were thawed and volumes equivalent to 15 µg of total protein were denatured with 0.05% RapiGest detergent (Waters, Milford, USA) and reduced with 5 mM dithiothreitol (Sigma-Aldrich, Oakville, Canada) at 60°C for 40 min. Alkylation was achieved with 15 mM iodoacetamide (Sigma-Aldrich) for 60 min in the dark at 22°C. A mixture of APOE heavy peptides was spiked into samples prior to addition of trypsin, while a mixture of 32 heavy peptides (30 candidates, ECM1, MBP) plus a heavy peptide for total APOE were spiked into the mixture after digestion, followed by addition of 1% trifluoroacetic acid. Digestion was carried out for 24 hours at 37°C with 1:30 trypsin-to-total protein ratio. Samples were then centrifuged at 1,000 g for 30 min and the supernatants retained. Peptides were purified using OMIX C18 tips, eluted in 4.5 µL of acetonitrile solution (65% acetonitrile, 0.1% formic acid) and finally diluted with 54 µL of water-formic acid mix (0.1% formic acid).

### Liquid chromatography-tandem mass spectrometry (LC-MS/MS)

Samples were analysed with a triple quadrupole mass spectrometer, TSQ Quantiva: (Thermo Scientific, San Jose, USA). Each sample (18 μL) was injected into an in-house-packed 3.3 cm pre-column (5 μm C18 particle, column inner diameter 150 μm), followed by a 15 cm analytical column (3 μm C18 particle, inner diameter 75 μm, tip diameter 8 μm). The liquid chromatography, EASY-nLC 1000 system (Thermo Fisher, Odense, Denmark) was coupled online to the TSQ Quantiva mass spectrometer with a nano-electrospray ionization source. A 37-min LC gradient was applied, with an increasing percentage of buffer B (0.1% formic acid in acetonitrile) for peptide elution at a flow rate of 300 nL/min. The SRM assay parameters were set up as follows: positive-ion mode, optimized collision energy values, adjusted dwell time, 0.7 Th Q1 resolution of full width at half-maximum and 0.7 Th in Q3 resolution. LC peaks for all peptides were manually inspected to ensure acquisition of minimum 10 points per LC peak. Raw data were uploaded and analyzed with Skyline software (University of Washington, Seattle, USA).

### Neuronal pentraxin receptor-1 (NPTXR) ELISA assay

We used the RayBio Human NPTXR ELISA kit, as recommended by the manufacturer (catalog # ELH-NPTXR, Ray Biotech, Norcross, GA, USA). All CSF samples were analyzed after a 25-fold dilution. For this independent validation, we used CSF samples from 12 AD patients, 21 patients with MCI and 23 control subjects. This cohort was used previously for mass spectrometric analyses, as outlined elsewhere ^19^. The samples were obtained by lumbar puncture and stored at -80°C until use. The Institutional Review Board of the Technical University of Munich approved the study and all patients signed an informed consent form.

### Clinical samples

Age and sex data were collected from all participants. In total, 101 CSF samples were retrospectively collected at the memory and dementia clinic of the 3rd Department of Neurology, “G. Papanikolaou”, School of Medicine, Aristotle University of Thessaloniki, Greece and from the Day Centers of the Greek Association of Alzheimer’s Disease and Related Disorders (GAARD), Thessaloniki, Greece. A summary of patient characteristics is shown in Table 1.

**Table 1.** Patient characteristics.

Set 1	Mild cognitive impairment	Mild AD dementia	Moderate AD dementia	Severe AD dementia
Participants, n	8	11	24	15
Age ^a^	75 (70.7, 80.5)	71 (68, 76.5)	76.5 (70.7, 78.25)	76 (69.5, 82)
Age ^b^	74.5 (7.8)	71.4 (8.4)	75.7 (6.4)	74.4 (9.3)
Sex-female, n (%)	3 (38)	3 (27)	13 (54)	6 (40)
MMSE ^a, c^	28 (26, 29)	24 (22, 25.5)	19 (16.8, 20)	8 (2.5, 10)
MMSE ^b^	27.6 (1.8)	23.9 (1.7)	18.5 (2.0)	6.5 (4.6)
Set 2				
Participants, n	6	8	16	13
Age ^a^	68 (60, 74)	76.5 (71.5, 80.7)	78.5 (74.7, 83.2)	75 (72, 76)
Age ^b^	67.6 (9.2)	76.2 (8.8)	78.1 (6.9)	71.1 (9.0)
Sex-female, n (%)	5 (83)	3 (38)	6 (38)	2 (15%)
MMSE ^a, c^	27.5 (26.2, 28.7)	24 (22.7, 24)	17.5 (16.7, 19)	7 (2, 10)
MMSE ^b^	27.7 (1.6)	23.6 (1.3)	17.6 (2.2)	6.2 (4.4)

AD, Alzheimer’s disease. ^a ^ Expressed as median (25 ^th^, 75 ^th^ percentile) ^b ^ Expressed as mean (standard deviation) ^c^ Mini-Mental State Examination

Patients suspected of having AD were examined by a specialist neuropsychiatrist and diagnosis was made based on the NINCDS/ADRDA criteria for probable AD ^20^. Disease severity was determined based on the Mini-Mental State Examination (MMSE) and clinical dementia rating (CDR) scores and patients were categorized as having mild (MMSE=20–26, CDR=1), moderate (MMSE=10–19, CDR=2) and severe (MMSE=0–9, CDR=3) dementia. Diagnosis of MCI was based on the description by Petersen, which is almost equivalent to the NIH-AA criteria for MCI due to AD ^21^. This study was approved by the GAARD scientific and ethics committees and by the Institutional Review Boards of Aristotle University and the University of Toronto. All participants signed an informed consent form.

A fraction of CSF samples were analyzed for core AD biomarkers (Aβ1-42, t-tau, p-tau) using an Innotest ELISA kit (Fujirebio Europe) ^22^. Overall, 54 participants were tested for Aβ1-42 (distributed by groups, MCI: n=10, mild: n=7, moderate: n=23, severe: n=14), 42 for t-tau (distributed by groups, MCI: n=9, mild: n=6, moderate: n=16, severe: n=11) and 43 for p-tau (distributed by groups, MCI: n=9, mild: n=5, moderate: n=21, severe: n=8).

All CSF samples were collected by lumbar puncture, inspected macroscopically for blood contamination, centrifuged and stored at -80°C in polypropylene tubes. Samples were shipped to the Lunenfeld−Tanenbaum Research Institute, Mount Sinai Hospital, Toronto, Canada and stored at -80°C until processing. Ethics approval was obtained from the Mount Sinai Hospital Research Ethics Board for use of these samples. 

### Data analysis

Clinical samples were randomized and ran in duplicate. The raw files were uploaded to Skyline software (version 3.5.0.9319), which was used for peak integration and quantification of the area under the curve (AUC). Relative quantification was performed as previously described ^16^. For peptides with amino acid methionine in the sequence, AUC _light_/AUC _heavy_ was calculated as: AUC (oxidized + non-oxidized) _light_/AUC (oxidized + non-oxidized) _heavy_. SRM data were manually evaluated and samples with poor integration were excluded. Identification of APOE phenotype was determined as described in our previous report ^18^.

### Statistical analysis

Statistical analysis was performed with R statistical and graphics software, version 3.5.0. Means, medians, standard deviations, interquartile ranges and coefficients of variations were calculated. Linear regression was used to test for differences in ages. For tests involving a dichotomous variable, such as sex, the Fisher’s exact test was used. Tests for differences in candidate protein abundance, MMSE score, CSF Aβ1-42, t-tau and p-tau across disease stages were adjusted by age and sex using multivariate linear regression. Correlation analyses for MMSE score and protein abundance were performed using Spearman’s rank correlation test. ROC curves were prepared for the most significant proteins and AUC values with 95% confidence intervals were calculated using the bootstrap method. AUC values were covariate-adjusted by age or sex when there was a significant association (p<0.05) between a marker and the covariates in controls ^23^. P-values for comparison between groups were reported as non-adjusted and adjusted for multiple comparison by the Holm method and p<0.05 was considered statistically significant.

## Results

### Patients’ characteristics

CSF samples from MCI and AD patients with different dementia severity (n=101) were randomized into two sets. The rationale for the randomization was to confirm the validity of our findings in separate assays, performed on different days. In the first set, 8 patients were diagnosed with MCI, 11 with mild, 24 with moderate and 15 with severe dementia, while in the second set, 6 patients had MCI, 8 mild, 16 moderate and 13 severe dementia ( Table 1).

**Figure 1.**
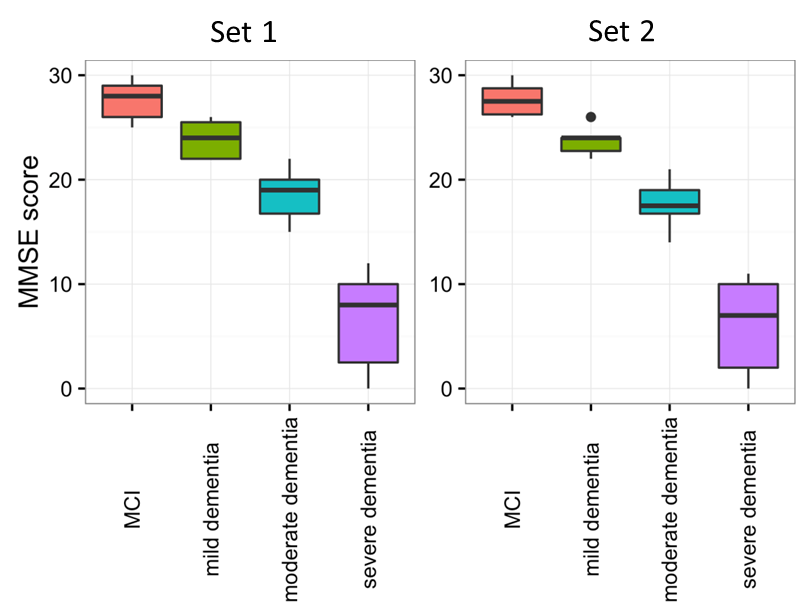
Distribution of cognitive test scores in mild cognitive impairment (MCI) and mild, moderate and severe Alzheimer’s disease (AD) dementia groups. The cognitive test Mini-Mental State Examination (MMSE) was compared between MCI, mild, moderate and severe AD dementia patients. A statistically significant difference in cognitive performance was observed among the four groups, in both sets (p<0.001). Horizontal lines represent medians. The number of patients per group is mentioned in Table 1.

The MMSE cognitive test was significantly different (p<0.001) in both sets between the four groups ( Figure 1, Table 1). As expected, MCI patients had the highest MMSE score, followed by mild, moderate, and severe AD. In the first set, the mean age (years) was 74.5 for MCI, 71.4 for mild, 75.7 for moderate and 74.4 for severe dementia. In the second set, the mean age (years) was 67.6 for MCI, 76.2 for mild, 78.2 for moderate and 71.1 for severe dementia. In set 1 there were 3 females each in the MCI and mild dementia groups, 13 in moderate and 6 in severe dementia groups, whereas in set 2, 5 females were in the MCI group, 3 in mild, 6 in moderate and 2 in severe dementia groups. Between groups, there was no difference in age (p=0.514) or sex (p=0.504) in set 1, while a small difference was found for age (p=0.041) and sex (p=0.047) between the four groups in set 2.

**Figure 2.**
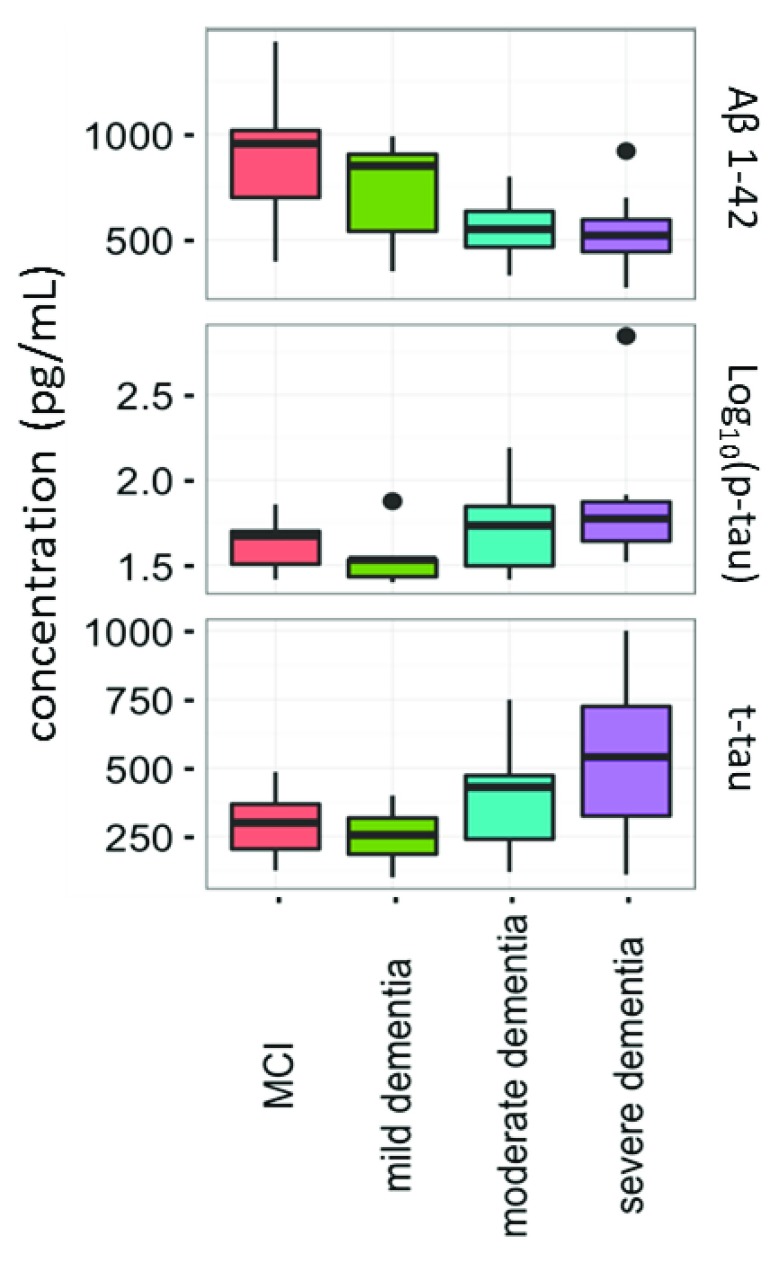
Distribution of core cerebrospinal fluid biomarkers Aβ1-42, t-tau and p-tau. The concentrations of these proteins were compared between mild cognitive impairment (MCI) (n=10) mild (n=7), moderate (n=23) and severe Alzheimer’s disease (n=14). Aβ1-42 and t-tau were significantly different between the tested groups (p<0.05). Horizontal lines represent medians.

Current CSF biomarkers were tested in a fraction of MCI, mild, moderate and severe AD patients. A statistical difference was observed between disease groups for Aβ1-42 (decreasing with severity) and t-tau (increasing with severity) (p<0.05); Aβ1-42 levels were differentially expressed between MCI *vs.* moderate AD dementia and MCI *vs.* severe AD dementia ( Supplementary Table 1). The distributions of Aβ1-42, t-tau and p-tau in the four groups are shown in Figure 2.

### Novel candidate biomarkers of AD patients

For evaluation of the 30 biomarker candidates, 101 CSF samples from MCI and AD patients were randomized into two separate sets.

Overall, the majority of the proteins showed similar distribution patterns across AD stages, with a trend towards a decline in CSF concentration with disease progression. Among all proteins, only NPTXR showed a statistically significant difference between MCI *vs.* combined moderate and severe AD groups in both sets of patients (set 1: p=0.004, set 2: p=0.039). The concentration of NPTXR decreased in advanced stages. However, this significance did not remain after multiple comparison correction by the Holm’s method.

Several other proteins also showed decreases in advanced stages of AD but did not consistently achieve statistical significance. In the first data set, proteins NPTXR, NPY and VGF were significantly different between the four groups (p=0.014, 0.033, 0.038, respectively), before correction for multiple comparison testing. After correction, the significance disappeared. Likewise, the findings observed in the first data set were not always-replicated in the second data set, but some proteins showed differential levels when comparing MCI *vs.* moderate and severe AD. These included BAI2, ECM1, FRRS1L, NPTXR, NPY, SLITRK1 and VGF (p=0.044, 0.033, 0.042, 0.004, 0.004, 0.048, 0.005 respectively). Proteins NPTXR, NPY and VGF were the most consistent, showing reductions in concentration with increasing AD severity.

Control protein ECM1 did not differ among MCI, mild, moderate and severe AD patients (set 1 p=0.200, set 2 p=0.926) but differed between MCI vs. moderate and severe AD groups, only in set 1. However when multiple correction was applied, the difference disappeared.

Statistical analysis of all candidates between the four groups and between MCI *vs.* moderate and severe AD dementia is shown in Supplementary Table 2.

**Figure 3.**
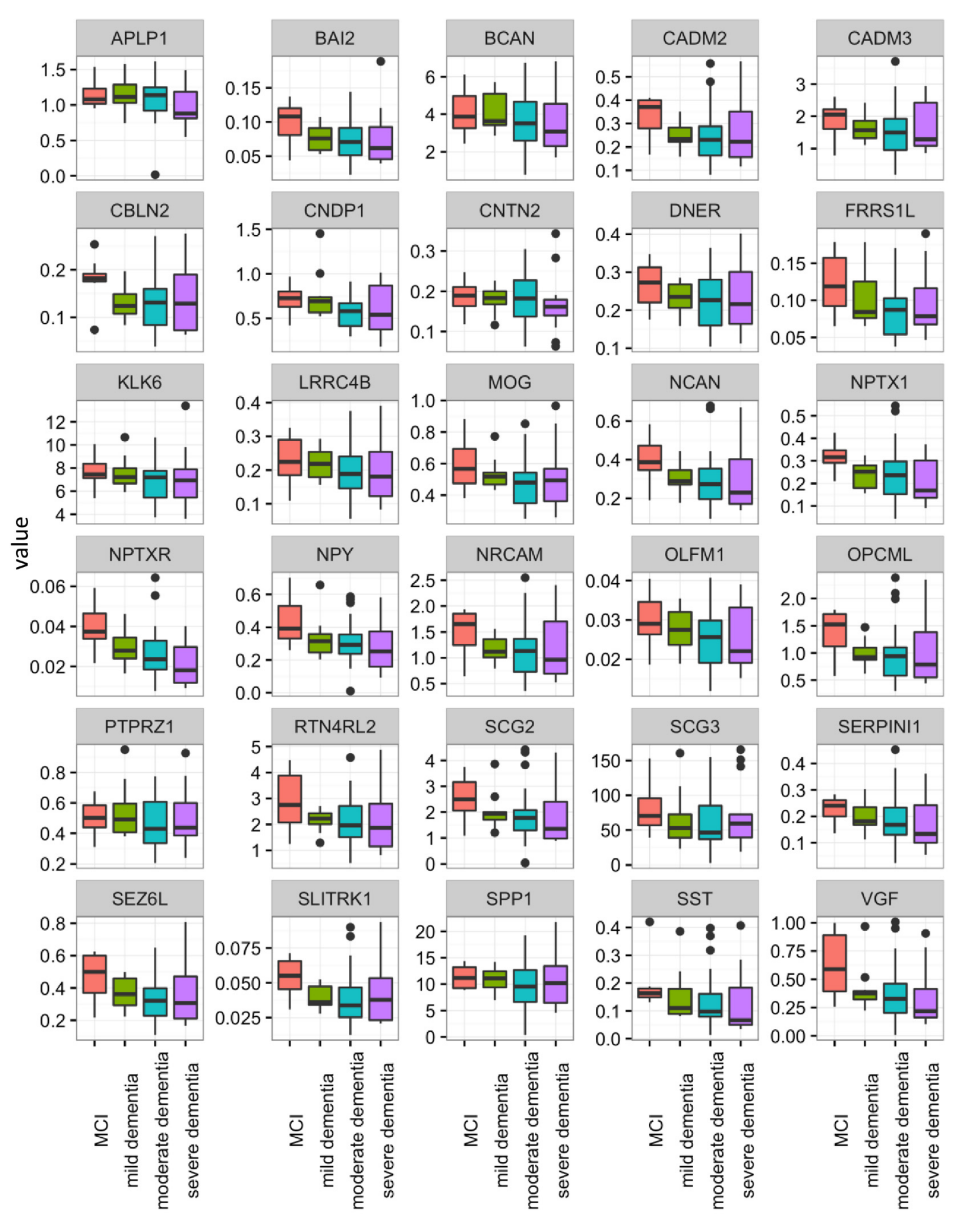
Distribution of candidate protein biomarkers in cerebrospinal fluid samples of set 1 (see text for definitions). Candidate proteins were measured with SRM assay and compared between mild cognitive impairment (MCI) (n=8), mild (n=11), moderate (n=24) and severe AD (n=15). Full gene names can be found in the website of the human gene nomenclature committee ( https://www.genenames.org/).

**Figure 4.**
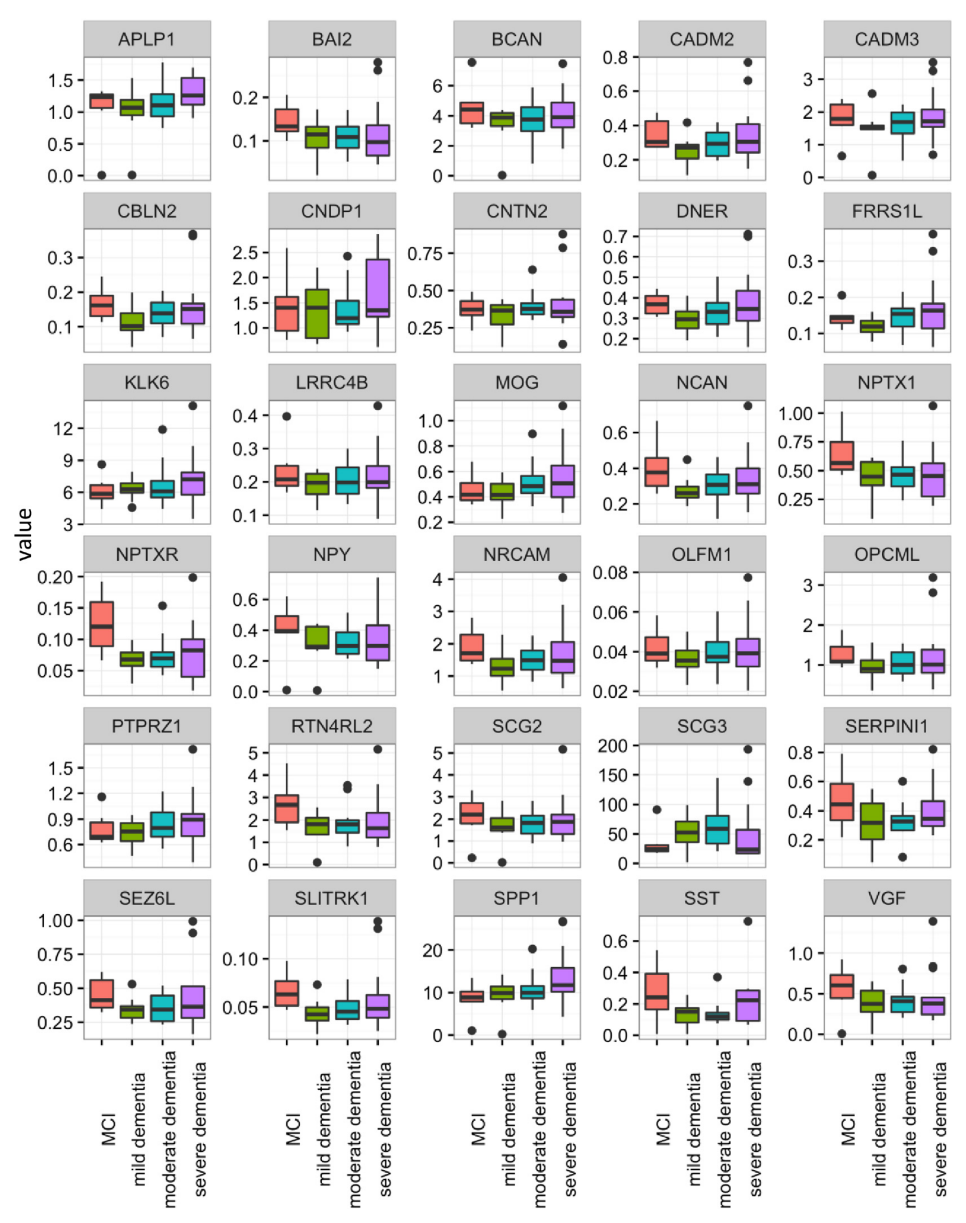
Distribution of candidate protein biomarkers in cerebrospinal fluid samples of set 2 (see text for definitions). Candidate proteins were measured with SRM assay and compared between mild cognitive impairment (MCI) (n=6), mild (n=8), moderate (n=16) and severe AD (n=13). Full gene names can be found in the website of the human gene nomenclature committee ( https://www.genenames.org/).

The reproducibility of the assays for control samples (pools of non-pathological CSFs) and clinical samples was <20% (data not shown). The distributions of all candidate proteins between the four disease groups in sets 1 and 2 are shown in Figure 3 and Figure 4.

### Diagnostic performance

**Figure 5.**
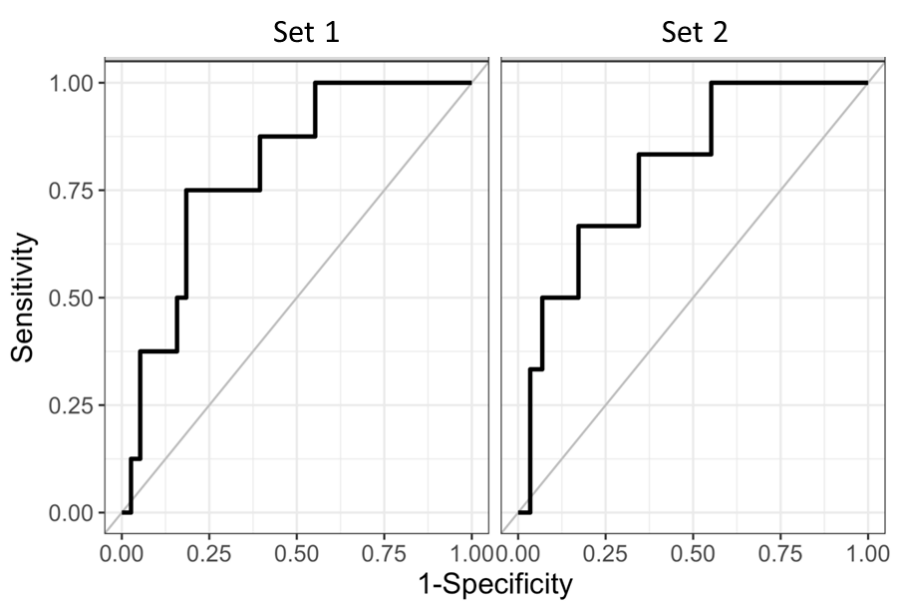
Receiver-operating characteristic (ROC) curves for the best performing candidate. ROC curve of NPTXR protein in set 1 and set 2; area under the curve value for set 1 was 0.799 (95% CI: 0.628, 0.928) and in set 2 was 0.799 (95% CI: 0.586, 0.960).

Diagnostic performance was evaluated by calculating the AUC for discriminating MCI *vs.* moderate and severe AD dementia. Based on the performance of candidates in both sets, only NPTXR protein showed a significant and reproducible separation between the two groups. In the first set, the AUC for NPTXR was 0.799 (95% CI: 0.628, 0.928) and in the second set was 0.799 (95% CI: 0.586, 0.960). Figure 5 shows ROC curves for this protein in both sets.

### Correlation of candidate proteins with MMSE score

Pairwise Spearman’s rank correlation was used to assess if there is a correlation between protein candidates and the cognitive test MMSE score. A few proteins showed a significant positive correlation with MMSE score ( Supplementary Table 3), which means that a lower score was associated with a lower protein concentration in CSF. Spearman’s rank correlation coefficients between level of these candidates and the cognitive test (for pairs significant at 0.05 level) was: 0.21 for BAI2, 0.23 for NCAN, 0.29 for NPY, 0.22 for OPCML, 0.29 for RTN4RL2, 0.26 for SCG2, 0.23 for SEZ6L, 0.25 for SST and 0.32 for VGF. The Spearman’s coefficient for NPTXR was 0.20 (not significant).

### Distribution of candidate proteins among APOE phenotypes

Overall, there was 31% APOE ε4 carriers among disease patients (14% among MCI, 21% among mild, 30% among moderate and 46% among severe AD dementia). APOE ε4 homozygous patients were present only in mild (n=2) and severe AD (n=2) groups. There was no significant difference in distribution of ε4 carriers between disease patients with different severity (p=0.138). Overall, five APOE phenotypes were identified in all subjects, ε2/ε3, ε2/ε4, ε3/ε3, ε3/ε4 and ε4/ε4, with no difference in the APOE phenotype frequencies among tested groups (p=0.160). In set 1 all five APOE phenotypes were present, ε2/ε3 (n=2), ε2/ε4 (n=1), ε3/ε3 (n=38), ε3/ε4 (n=13) and ε4/ε4 (n=4), while in set 2 only three: ε2/ε3 (n=3), ε3/ε3 (n=27), ε3/ε4 (n=13). The frequencies of APOE phenotypes are shown in Table 2.

**Table 2.** APOE phenotype distribution.

APOE phenotype	Mild cognitive impairment	Mild AD dementia	Moderate AD dementia	Severe AD dementia	Total
ε4-carriers (%)	14	21	30	46	31
ε2/ε3	0	2	2	1	5
ε2/ε4	0	0	1	0	1
ε3/ε3	12	13	26	14	65
ε3/ε4	2	2	11	11	26
ε4/ε4	0	2	0	2	4
**Grand total**	**14**	**19**	**40**	**28**	**101**

APOE, apolipoprotein E; AD, Alzheimer’s disease.

In both set of samples, none of the proteins showed a reproducible difference in abundance between APOE phenotypes (data not shown). Only FRRS1L protein showed a modest significance and only in the first set (p=0.040, when not adjusted for multiple comparison). There was no difference in proteins in set 2 between different phenotypes (p>0.05).

### Validation of NPTXR in CSF by ELISA

**Figure 6.**
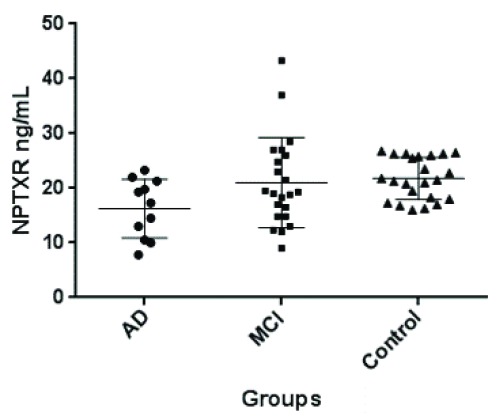
Distribution of CSF NPTXR concentrations, as measured by ELISA, in CSF of patients with Alzheimer’s disease (AD; n=12), mild cognitive impairment (MCI; n=21) and controls (n=23). The differences were statistically significant only between controls and AD patients by Mann-Whitney test (p=0.004). Horizontal lines represent means and 25–75 percentiles. This cohort has been described elsewhere ^19^. For more details see text.

In order to validate our findings of decreased NPTXR in CSF of MCI and AD patients, we analyze CSF NPTXR by sandwich ELISA assay. For this independent validation, we used CSF samples from 12 AD patients, 21 patients with MCI and 23 control subjects. The results of CSF NPTXR concentration in the three groups of patients are shown in Figure 6. Controls had the highest level, followed by MCI and AD. The differences between controls and MCI were not statistically significant by the Mann-Whitney non-parametric test (p=0.52). Also, the differences were not significant between MCI and AD patients (p=0.10). However, the differences between controls and AD were highly significant (p=0.004). These results further support our hypothesis that NPTXR is a new CSF biomarker of AD, decreasing progressively with disease severity.

Dataset 1. Raw data for the results included in this study http://dx.doi.org/10.5256/f1000research.15095.d208304
Copyright: © 2018 Begcevic I et al. 2018 Data associated with the article are available under the terms of the Creative Commons Zero "No rights reserved" data waiver (CC0 1.0 Public domain dedication).

## Discussion

The main objective of the present study was to evaluate 30 brain-related proteins as CSF biomarkers of AD severity and progression. These highly specific brain proteins were assessed in AD patients with different stages, including MCI, mild, moderate and severe AD dementia. Protein NPTXR showed potential as a biomarker of disease progression. Significant and consistent differences in CSF NPTXR levels were observed between MCI *vs.* combined moderate and severe AD dementia groups.

NPTXR protein is a member of the neuronal pentraxin family, expressed predominately in the brain, with the highest expression observed in the hippocampus and cerebellum ^24^. This transmembrane presynaptic protein was suggested to be involved in the activation of both excitatory and inhibitory neurons ^25^. NPTXR has been suggested previously as a potential prognostic biomarker ^26^, more specific for AD, compared to Parkinson’s disease ^27^. Differential abundance of NPTXR has been observed in asymptomatic carriers of AD familial mutations, compared to non-carriers, with elevated levels observed in asymptomatic carriers ^28^. Wildsmith *et al*. observed a similar trend of NPTXR abundance between MCI and AD groups, as found here; i.e. increased levels in the MCI group, with AD patients having lower levels ^26^. A decreasing abundance of NPTXR was also observed in longitudinally followed AD patients. This data are in accord with our study, since NPTXR levels declined proportionally with advanced AD dementia stages. Additional accumulating evidence by Hendrickson *et al*. further suggests that NPTXR represents a new biomarker of AD disease progression, decreasing with the severity of AD ^29^. The latter study identified VGF as an additional progression biomarker, as we also found here.

The observed pattern of decline in abundance with disease severity for a few proteins, in addition to NPTXR, could be partially explained by their brain-specificity. Since our candidates were selected to have high specificity for brain tissue expression, these proteins could be expressed throughout various brain regions and their decrease may represent the overall decline in cortical volume, thus serving as markers of brain atrophy. This suggestion could be confirmed in future studies, by comparing brain imaging data with abundance of specific proteins in CSF. The superiority of NPTXR over other proteins may be related to its high expression in the hippocampus.

APOE ε4 allele represents the main genetic risk factor for developing AD. Carriers for APOE ε4 have earlier age of disease onset and more pronounced amyloid pathology compared to non-carriers. For example, amyloid plaques are more abundant in ε4 carriers ^30^, with lower CSF concentration of Aβ1-42 in AD patients ^31^. In addition to enhanced Aβ plaques in the brain, ε4 carriers exhibit vascular Aβ deposition ^31^. Therefore, we aimed to evaluate if levels of our candidate biomarkers change with APOE phenotype. In this study, none of the candidates showed any reproducible difference in abundance between APOE phenotypes. However, this needs to be further investigated due to our limited sample size and considering that not all APOE phenotypes were identified in both set of samples.

Some limitations of our study are associated with the selection of the 30 brain-specific proteins. Our focus was on predicted secreted and membrane-bound proteins, since the majority of HPA brain-enriched proteins are membrane and/or secreted ^15^ and most of the CSF proteins are membrane-bound or secreted ^32^. Intracellular proteins, which were excluded from this study, may have lower abundance in normal CSF but under pathological conditions they could be released into the CSF. Other limitations are related to the patients included in the study. Our cohorts did not include preclinical AD, which would allow assessment of the proposed candidates from the very early stage of developing AD. Cognitively healthy, age-matched controls were not included in this study since we aimed to test biomarkers in different stages of disease progression. Moreover, only a subset of patients had information on current AD biomarkers (Aβ1-42, t-tau, p-tau). Therefore, the diagnostic accuracy (ROC curve analysis) of NPTXR was not compared to the existing AD CSF biomarkers.

Our candidate biomarkers should be further tested in individuals encompassing the whole AD continuum, from preclinical to more advanced clinical stages. Preferentially, longitudinally followed patients should be monitored to assess the prognostic potential of the candidates, over sufficient period of time, allowing disease progression to the next stage. The approximate annual rate of progression of MCI to AD dementia is 10 to 15% ^33^.

In summary, in this study, we evaluated 30 brain-specific proteins as candidate CSF biomarkers of AD severity, utilizing multiplex mass spectrometry-based quantification. The protein NPTXR showed the most promise as a potential biomarker of disease progression. Interestingly, at least two other previous studies have also identified NPTXR as a highly promising biomarker of progression of AD. CSF NPTXR levels decline proportionally, as AD becomes more severe. This finding needs to be validated in a larger, longitudinally followed cohort. We suggest that NPTXR may have value as a CSF biomarker for assessing the efficacy of new therapies for AD.

## Data availability

The data referenced by this article are under copyright with the following copyright statement: Copyright: © 2018 Begcevic I et al.

Data associated with the article are available under the terms of the Creative Commons Zero "No rights reserved" data waiver (CC0 1.0 Public domain dedication). 

Dataset 1: Raw data for the results included in this study. DOI, http://dx.doi.org/10.5256/f1000research.15095.d208304 ^34^.

## Supplementary material

Supplementary Table 1: Statistical analysis between four patient groups for Aβ1-42, t-tau and p-tau.

Supplementary Table 2: Statistical analysis of candidates between four patient groups and MCI *vs.* moderate and severe AD dementia.

Supplementary Table 3: Spearman’s rank correlation between candidate levels and MMSE score.

## References

[1] Ballard C Gauthier S Corbett A: Alzheimer's disease. *Lancet.* 2011;377(9770):1019–1031. 10.1016/S0140-6736(10)61349-9 21371747

[2] McKhann GM Knopman DS Chertkow H: The diagnosis of dementia due to Alzheimer's disease: Recommendations from the National Institute on Aging-Alzheimer's Association workgroups on diagnostic guidelines for Alzheimer's disease. *Alzheimers Dement.* 2011;7(3):263–269. 10.1016/j.jalz.2011.03.005 21514250 PMC3312024

[3] Nakamura A Kaneko N Villemagne VL: High performance plasma amyloid-β biomarkers for Alzheimer's disease. *Nature.* 2018;554(4691):249–54. 10.1038/nature25456 29420472

[4] Jack CR Jr Albert MS Knopman DS: Introduction to the recommendations from the National Institute on Aging-Alzheimer's Association workgroups on diagnostic guidelines for Alzheimer's disease. *Alzheimers Dement.* 2011;7(3):257–262. 10.1016/j.jalz.2011.03.004 21514247 PMC3096735

[5] WHO: Dementia: a public health priority. WHO.2012 Reference Source

[6] Kroksveen AC Opsahl JA Aye TT: Proteomics of human cerebrospinal fluid: discovery and verification of biomarker candidates in neurodegenerative diseases using quantitative proteomics. *J Proteomics.* 2011;74(4):371–388. 10.1016/j.jprot.2010.11.010 21111852

[7] Blennow K Hampel H Weiner M: Cerebrospinal fluid and plasma biomarkers in Alzheimer disease. *Nat Rev Neurol.* 2010;6(3):131–144. 10.1038/nrneurol.2010.4 20157306

[8] Blennow K de Leon MJ Zetterberg H: Alzheimer's disease. *Lancet.* 2006;368(9533):387–403. 10.1016/S0140-6736(06)69113-7 16876668

[9] Khan TK Alkon DL: Alzheimer's Disease Cerebrospinal Fluid and Neuroimaging Biomarkers: Diagnostic Accuracy and Relationship to Drug Efficacy. *J Alzheimers Dis.* 2015;46(4):817–836. 10.3233/JAD-150238 26402622

[10] Sunderland T Wolozin B Galasko D: Longitudinal stability of CSF tau levels in Alzheimer patients. *Biol Psychiatry.* 1999;46(6):750–755. 10.1016/S0006-3223(99)00143-2 10494442

[11] Williams JH Wilcock GK Seeburger J: Non-linear relationships of cerebrospinal fluid biomarker levels with cognitive function: an observational study. *Alzheimers Res Ther.* 2011;3(1):5. 10.1186/alzrt64 21329517 PMC3109414

[12] Drabovich AP Dimitromanolakis A Saraon P: Differential diagnosis of azoospermia with proteomic biomarkers ECM1 and TEX101 quantified in seminal plasma. *Sci Transl Med.* 2013;5(212):212ra160. 10.1126/scitranslmed.3006260 24259048

[13] Martinez-Morillo E Garcia Hernandez P Begcevic I: Identification of novel biomarkers of brain damage in patients with hemorrhagic stroke by integrating bioinformatics and mass spectrometry-based proteomics. *J Proteome Res.* 2014;13(2):969–981. 10.1021/pr401111h 24295473

[14] Begcevic I Brinc D Drabovich AP: Identification of brain-enriched proteins in the cerebrospinal fluid proteome by LC-MS/MS profiling and mining of the Human Protein Atlas. *Clin Proteomics.* 2016;13:11. 10.1186/s12014-016-9111-3 27186164 PMC4868024

[15] Uhlén M Fagerberg L Hallström BM: Proteomics. Tissue-based map of the human proteome. *Science.* 2015;347(6220):1260419. 10.1126/science.1260419 25613900

[16] Begcevic I Brinc D Dukic L: Targeted mass spectrometry-based assays for relative quantification of 30 brain-related proteins and their clinical applications. *J Proteome Res.* 2018. 10.1021/acs.jproteome.7b00768 29708756

[17] Liu CC Liu CC Kanekiyo T: Apolipoprotein E and Alzheimer disease: risk, mechanisms and therapy. *Nat Rev Neurol.* 2013;9(2):106–118. 10.1038/nrneurol.2012.263 23296339 PMC3726719

[18] Martínez-Morillo E Nielsen HM Batruch I: Assessment of peptide chemical modifications on the development of an accurate and precise multiplex selected reaction monitoring assay for apolipoprotein e isoforms. *J Proteome Res.* 2014;13(2):1077–1087. 10.1021/pr401060x 24392642

[19] Begcevic I Brinc D Brown M: Brain-related proteins as potential CSF biomarkers of Alzheimer’s disease: A targeted mass spectrometry approach. *J Proteomics.* 2018;182:12–20. 10.1016/j.jprot.2018.04.027 29684683

[20] McKhann G Drachman D Folstein M: Clinical diagnosis of Alzheimer's disease: report of the NINCDS-ADRDA Work Group under the auspices of Department of Health and Human Services Task Force on Alzheimer's Disease. *Neurology.* 1984;34(7):939–944. 10.1212/WNL.34.7.939 6610841

[21] Petersen RC Smith GE Waring SC: Mild cognitive impairment: clinical characterization and outcome. *Arch Neurol.* 1999;56(3):303–308. 10.1001/archneur.56.3.303 10190820

[22] Toledo JB Zetterberg H van Harten AC: Alzheimer's disease cerebrospinal fluid biomarker in cognitively normal subjects. *Brain.* 2015;138(Pt 9):2701–15. 10.1093/brain/awv199 26220940 PMC4643624

[23] Janes H Longton G Pepe M: Accommodating Covariates in ROC Analysis. *Stata J.* 2009;9(1):17–39. 20046933 PMC2758790

[24] Dodds DC Omeis IA Cushman SJ: Neuronal pentraxin receptor, a novel putative integral membrane pentraxin that interacts with neuronal pentraxin 1 and 2 and taipoxin-associated calcium-binding protein 49. *J Biol Chem.* 1997;272(34):21488–21494. 10.1074/jbc.272.34.21488 9261167

[25] Lee SJ Wei M Zhang C: Presynaptic Neuronal Pentraxin Receptor Organizes Excitatory and Inhibitory Synapses. *J Neurosci.* 2017;37(5):1062–1080. 10.1523/JNEUROSCI.2768-16.2016 27986928 PMC5296791

[26] Wildsmith KR Schauer SP Smith AM: Identification of longitudinally dynamic biomarkers in Alzheimer's disease cerebrospinal fluid by targeted proteomics. *Mol Neurodegener.* 2014;9:22. 10.1186/1750-1326-9-22 24902845 PMC4061120

[27] Yin GN Lee HW Cho JY: Neuronal pentraxin receptor in cerebrospinal fluid as a potential biomarker for neurodegenerative diseases. *Brain Res.* 2009;1265:158–170. 10.1016/j.brainres.2009.01.058 19368810

[28] Ringman JM Schulman H Becker C: Proteomic changes in cerebrospinal fluid of presymptomatic and affected persons carrying familial Alzheimer disease mutations. *Arch Neurol.* 2012;69(1):96–104. 10.1001/archneurol.2011.642 22232349 PMC3632731

[29] Hendrickson RC Lee AY Song Q: High Resolution Discovery Proteomics Reveals Candidate Disease Progression Markers of Alzheimer's Disease in Human Cerebrospinal Fluid. *PLoS One.* 2015;10(8):e0135365. 10.1371/journal.pone.0135365 26270474 PMC4535975

[30] Schmechel DE Saunders AM Strittmatter WJ: Increased amyloid beta-peptide deposition in cerebral cortex as a consequence of apolipoprotein E genotype in late-onset Alzheimer disease. *Proc Natl Acad Sci U S A.* 1993;90(20):9649–9653. 10.1073/pnas.90.20.9649 8415756 PMC47627

[31] Prince JA Zetterberg H Andreasen N: *APOE* epsilon4 allele is associated with reduced cerebrospinal fluid levels of Abeta42. *Neurology.* 2004;62(11):2116–2118. 10.1212/01.WNL.0000128088.08695.05 15184629

[32] Zhang Y Guo Z Zou L: A comprehensive map and functional annotation of the normal human cerebrospinal fluid proteome. *J Proteomics.* 2015;119:90–99. 10.1016/j.jprot.2015.01.017 25661039

[33] Hansson O Zetterberg H Buchhave P: Association between CSF biomarkers and incipient Alzheimer's disease in patients with mild cognitive impairment: a follow-up study. *Lancet Neurol.* 2006;5(3):228–234. 10.1016/S1474-4422(06)70355-6 16488378

[34] Begcevic I Tsolaki M Brinc D: Dataset 1 in: Neuronal pentraxin receptor-1 is a new cerebrospinal fluid biomarker of Alzheimer’s disease progression. *F1000Research.* 2018 10.5256/f1000research.15095.d208304 PMC6081984 30191060

