# Supplementary material for: Neuronal pentraxin receptor-1 is a new cerebrospinal fluid biomarker of Alzheimer’s disease progression
Source: F1000Res. 2018 Jul 5;7:1012. [Version 1] doi: 10.12688/f1000research.15095.1 (PMC6081984; doi:10.12688/f1000research.15095.1)
Supplement: Peer review history

# Peer review: Referee response for version 1

**DOI:** 10.5256/f1000research.16440.r36772

**Published:** 2018-08-15

**Authors:** Mohd M. Khan

**Affiliations:** 1 National Institute of Allergy and Infectious Diseases, National Institutes of Health, Bethesda, MD, 20892, USA

In the US alone, Alzheimer’s disease (AD) is the 6th leading cause of death. Unfortunately there are no clinical biomarkers of AD that could be used to efficiently and successfully diagnose the disease onset, monitor the AD progression, and evaluate the efficiency of therapeutic interventions against AD. According to Alzheimer's association, early and accurate AD diagnosis could save $7.9 trillion in hospital-associated costs. Hence to fill this diagnostic gap, present work aims to identify and validate AD biomarkers in cerebrospinal fluid (CSF) patient samples using mass spectrometry-based targeted proteomics. The manuscript is well written and methods are either sufficiently described and/or appropriate earlier works are cited (particularly for targeted method development and protein quantitation). However I suggest authors should consider submitting the raw MS data to ProteomeXchange if not done already.

 Targeted proteomics was used to quantify 30 candidate proteins in CSF samples obtained from 101 patients that had mild to severe AD as well as patients with mild cognitive impairment (MCI). In both the sets, 28 severe AD, 40 moderate AD, 19 mild AD, and 14 MCI patient samples were quantified for candidate biomarkers using SRM method. One biomarker candidate of interest neuronal pentraxin receptor-1 (NPTXR), quantified by mass spectrometry and validated in a separate set by ELISA, was found useful in differentiating AD from control samples. Severe AD patients had lower levels of NPTXR in CSF than those in control CSF samples; in contrast, considerable variation in the levels of NPTXR levels in MCI patients was observed. However the rationale behind not segregating samples in 5 groups (control, MCI, mild-, moderate-, and severe-dementia) for ELISA validation is missing and could be added. For the NPTXR validation, authors could have done secondary validation of NPTXR levels in present cohort (large group; n=101) followed by an independent validation in a separate cohort as done in Figure 6 (n=56). Nonetheless this early work on AD biomarker discovery is interesting and a longitudinal study will be helpful in establishing NPTXR as a true biomarker of AD progression and disease severity.

I have read this submission. I believe that I have an appropriate level of expertise to confirm that it is of an acceptable scientific standard.

# Peer review: Referee response for version 1

**DOI:** 10.5256/f1000research.16440.r36375

**Published:** 2018-08-08

**Authors:** Julie Shaw

**Affiliations:** 1 Department of Pathology and Laboratory Medicine, The Ottawa Hospital, Eastern Ontario Regional Laboratories Association, University of Ottawa, Ottawa, ON, Canada

Currently there are no biomarkers for AD used in clinical practice. Current methods for evaluating patients only allow for diagnoses of probable AD while definitive diagnosis can only take place at the time of post-mortem examination.  This study aims to identify much needed biomarkers for AD in CSF using mass spectrometry. The authors used tandem mass spectrometry to measure the concentrations of 30 CSF-specific proteins, which they previously identified, in patient specimens.  The patient cohort included those with mild cognitive impairment, mild, moderate and severe AD, as determined by the MMSE and CDR ratings.  The authors identified one candidate protein, neuronal pentraxin receptor -1 (NPTXR), as promising in its ability to differentiate MCI from moderate and severe AD.  Independent assessment of NPTXR concentrations in patient specimens using an ELISA confirmed that concentrations of this protein are lower in CSF from patients with moderate and severe AD compared to control patients.   

 This manuscript is well written and I only have minor comments, which I will outline below. 

 In the results section, the authors state that the reproducibility of the assays for control samples and clinical samples was <20%.  It is unclear to me what this refers to and it would be helpful for the authors to clarify, especially since the data are not shown.

 How was cognitive level assessed for the patient specimens used for the ELISA measurements?  It appears that these specimens came from a different institution than those used for the mass spectrometry analysis. If a different assessment scheme was employed for these specimens, this could be confounding. I would suggest that the authors comment on this.

I have read this submission. I believe that I have an appropriate level of expertise to confirm that it is of an acceptable scientific standard.

# Peer review: Referee response for version 1

**DOI:** 10.5256/f1000research.16440.r36373

**Published:** 2018-08-06

**Authors:** Edward W. Randell

**Affiliations:** 1 Department of Laboratory Medicine, Faculty of Medicine, Memorial University of Newfoundland, St. John's, NL, Canada

There are currently no blood based or CSF biomarkers commonly used on a routine basis for screening, diagnosis, or monitoring progression of Alzheimer’s disease. This work is an effort to fill the gap as it examines 30 candidate proteins from CSF and their relationship with disease progression. This was done by examining levels of the CSF proteins in patients at various stages of disease ranging from mild cognitive impairment to severe dementia. Initial assay of the 30 proteins was carried out using tryptic digestion and analysis of signature peptides by LC-MS/MS selective reaction monitoring technique. An attempt to validate results was undertaken by examination in a second set of patients representing the four stages of disease, from mild cognitive impairment to mild, moderate, and severe dementia. The study shows that the biomarker, neuronal pentraxin receptor-1 best discriminates mild cognitive impairment from advanced stages of the disease. This finding was confirmed by ELISA assay of the same protein. The study concludes that this CSF protein in a potential biomarker of Alzheimer’s disease progression with potential utility in monitoring treatment. The patient population was well described, and the experimental design sound. Findings of the study are well discussed with previous work and with limitations identified.

 Although, not explicitly stated this study apparently measures the 30 candidate biomarkers in CSF from 101 different individuals at different disease stages. Future use of neuronal pentraxin receptor-1, or any biomarker, for disease monitoring purposes at least partially involves establishing a baseline for individual patients and possible serial measurement. Based on information in the box and whisker plots, results for neuronal pentraxin receptor-1 in patients with mild cognitive impairment varied over a range that was about 3-fold and showed significant overlap with results of patients from the moderate and severe dementia groups. There were also noted outliers in the latter two groups (moderate and severe) showing results well within and even higher than in patients with mild cognitive impairment. Moreover, most of the biomarkers examined showed similar outliers in both sets of analyses. This presents many questions. It is not clear is these outliers for the different biomarkers are from the same patients (or samples) or if different patients and samples produce different biomarker outliers. Also, it is not clear if the neuronal pentraxin receptor-1 outliers, which represent 5 to 10% of the samples from patients with moderate and severe disease, are causes by pre-analytical issues concerning samples or confounding physiological/pathological processes in these patients. It is also not clear how variable levels of this protein is in CSF from an individual patient; and the imprecision of the two different assays (LC-MS/MS method and ELISA) for neuronal pentraxin receptor-1 used was not stated. Between-run assay imprecision and intra-individual biological variability will have bearing on the usefulness of a CSF neuronal pentraxin receptor-1 measurement in practice. It would also be interesting to see how levels change in individual patients as Alzheimer’s disease progresses. But, the requirement of repeated lumbar puncture to assess disease course using this or any CSF biomarkers will be a difficult sell given the absence of effective disease modifying therapy. Nevertheless, this does not take away from the value of this early work in highlighting potential value of neuronal pentraxin receptor-1 for disease progression with potential future clinical value if effective disease modifying treatments become available. But as implied by the authors, what would be most useful is a biomarker, like neuronal pentraxin receptor-1, that not only correlates with disease severity but is also predictive of Alzheimer’s disease progression and related outcomes.

I have read this submission. I believe that I have an appropriate level of expertise to confirm that it is of an acceptable scientific standard.

# Peer review: Referee response for version 1

**DOI:** 10.5256/f1000research.16440.r36054

**Published:** 2018-07-26

**Authors:** Georgios Pampalakis

**Affiliations:** 1 Department of Pharmacy, School of Health Sciences, University of Patras, Patras, Greece

AD is the most common neurodegenerative diseases. Unfortunately, there no molecular biomarkers for the disease yet. In the present study the authors used a well described cohort of AD patients to identify new protein-based biomarkers in CSF that can determine the progression of AD. The procedure was based on identifying potential protein biomarkers from Human Protein Atlas (30 brain-specific proteins were selected) and design analytical method based on MS for their determination in CSF. After careful examination of clinical specimens, the authors identified a new biomarker that was validated with ELISA, the NPTXR. The authors did not find any correlation of the potential biomarkers tested (including NPTXR) with the status of ApoE polymorphism. Finally, the limitations of their study are well-described. A major limitation as noted is the absence of normal samples for the analysis of the potential biomarkers. Although not entirely required for this study, analysis of normal CSF samples will help to identify biomarkers for AD diagnosis. However, the authors validated NPTXR in control samples with ELISA assay and found not statistically different levels between controls and patients with mild cognitive impairment. In conclusion, the present study is well-designed and adds new information on the development of new AD biomarker. It will worth in future studies to examine the levels of NPTXR in other neurodegerative diseases such as Parksinson to determine the specificity.

I have read this submission. I believe that I have an appropriate level of expertise to confirm that it is of an acceptable scientific standard.
